# High-Throughput and Computational Study of Leaf Senescence through a Phenomic Approach

**DOI:** 10.3389/fpls.2017.00250

**Published:** 2017-02-23

**Authors:** Jae IL Lyu, Seung Hee Baek, Sukjoon Jung, Hyosub Chu, Hong Gil Nam, Jeongsik Kim, Pyung Ok Lim

**Affiliations:** ^1^Center for Plant Aging Research, Institute for Basic ScienceDaegu, South Korea; ^2^Department of New Biology, Daegu Gyeongbuk Institute of Science and TechnologyDaegu, South Korea

**Keywords:** time-series analysis, leaf senescence, lifespan, life history, high-throughput phenotyping, phenome, *Arabidopsis*

## Abstract

Leaf senescence is influenced by its life history, comprising a series of developmental and physiological experiences. Exploration of the biological principles underlying leaf lifespan and senescence requires a schema to trace leaf phenotypes, based on the interaction of genetic and environmental factors. We developed a new approach and concept that will facilitate systemic biological understanding of leaf lifespan and senescence, utilizing the phenome high-throughput investigator (PHI) with a single-leaf-basis phenotyping platform. Our pilot tests showed empirical evidence for the feasibility of PHI for quantitative measurement of leaf senescence responses and improved performance in order to dissect the progression of senescence triggered by different senescence-inducing factors as well as genetic mutations. Such an establishment enables new perspectives to be proposed, which will be challenged for enhancing our fundamental understanding on the complex process of leaf senescence. We further envision that integration of phenomic data with other multi-omics data obtained from transcriptomic, proteomic, and metabolic studies will enable us to address the underlying principles of senescence, passing through different layers of information from molecule to organism.

## Introduction

Leaf senescence, although a degenerative cellular process, is finely regulated and occurs by an intricate integration of multiple developmental and environmental signals. As a consequence, it is assumed that leaf senescence is a highly complex process involving the collective actions of thousands of genes and multiple pathways associated with aging, as well as their interplays, thereby complicating genetic and molecular analyses of senescence (Buchanan-Wollaston et al., 2005; Breeze et al., 2011; Schippers, 2015; Li et al., 2016; Liebsch and Keech, 2016; Woo et al., 2016). Indeed, conventional molecular and genetic approaches in which one gene or mutant at a time is identified and characterized have been shown to be limited for revealing the global picture of molecular programs involved in leaf senescence (Guo, 2013; Kim et al., 2016). An additional pitfall experienced in previous studies is that leaf senescence has been achieved through a limited set of phenotypes in a narrow temporal window of senescence, mostly at an aged stage (Thomas, 2013).

The recent advances within omics technologies, including genomics, transcriptomics, proteomics, and metabolomics, have facilitated open innovation strategies toward systematic understanding of complex questions of plant growth, development, and responses to environments (Mochida and Shinozaki, 2011; Humplík et al., 2015; Rajasundaram and Selbig, 2016). However, the high-throughput phenotyping technologies for analyses of total physiological traits in plants lag behind our ability to investigate molecular omics, although measurement of physiological responses has been recognized as being essential to determine the implications of their reactions or responses (Furbank and Tester, 2011). One of the current technical challenges is therefore to advance the phenotyping system to allow numerous phenotypic analyses in an automated and high-throughput manner for a large set of plant populations under various conditions over time (Yang et al., 2013; Rahaman et al., 2015). These efforts are also being extended to address specific questions by establishing the phenotyping pipeline with specialized and sophisticated experimental designs and tools (Topp et al., 2013; Crowell et al., 2014; Slovak et al., 2014).

**FIGURE 1:**
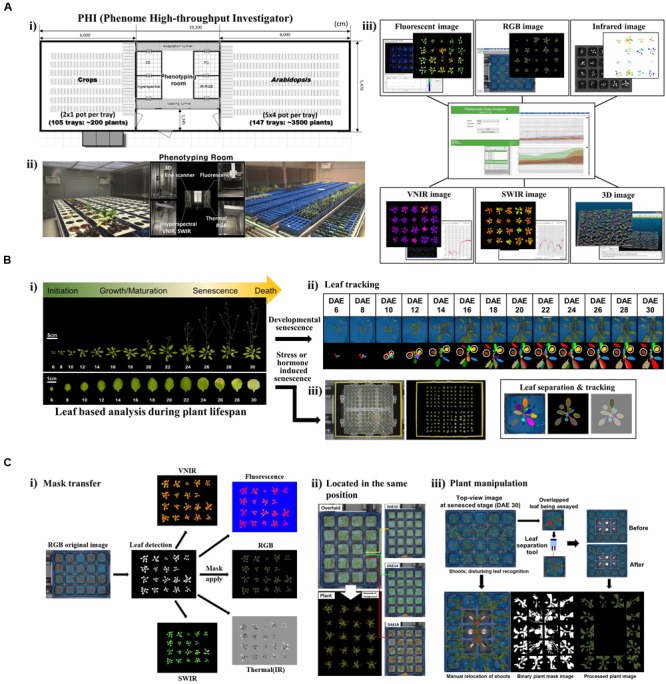
**Scheme of the PHI and its application in leaf senescence research (A)** Overview of the PHI system. **(i)** The PHI is an automated high-throughput phenotyping system coupled with a controlled plant growth system. The image station is equipped with five non-invasive camera-based imaging units: fluorescent, RGB [top and side views (line scanner)], infrared, hyperspectral (VNIR; 400 to 1,000 nm, SWIR; 1,000 to 2,500 nm), and three-dimensional (3D) imaging. **(ii)** Two plant growth rooms supported the growth of different types of plants with an automatic and precise control of the environment. **(iii)** Screenshot of the PHI image analyzer software and image data. **(B)** Leaf-based analysis during plant lifespan. **(i)** Life cycles of leaf organs and entire plants in *Arabidopsis*. Shown are representative *Arabidopsis* plants and leaves when the age of the third rosette leave is within DAE 6 to DAE 30. Responses of leaf senescence in *Arabidopsis* were assessed using developmental **(ii)** and stress- or hormone-induced **(iii)** senescence assays. **(ii)** Developmental senescence was monitored using leaf tracking and leaf separation. A representative plant with the third and fourth leaves of DAE 6 through DAE 30 in the pot where blue felt was placed on the top of the soil (Upper), and the pseudo-color image indicates correct recognition of individual leaves from plants (Lower). The third (brown) and fourth (yellow) leaves used for the senescence assay are marked with circles. **(iii)** Stress- or hormone-induced senescence was monitored using 24-well plates containing stress-inducing chemicals or hormones. **(C)** Leaf tracking for *in planta* senescence assay. **(i)** RGB image-based plant mask generation and its utilization in other image analyses. **(ii)** Location of a tray in the same position. Overlays of time-series images of a tray taken at DAE 10, 14, and 18 (Top, Middle, and Bottom of the Right panel). Overlaid original image (Left top) and plant image (Left bottom). **(iii)** Special manipulation of plants when assaying developmental senescence. Top-view RGB image at the senesced stage (DAE 30) encountered problems with the main or axillary shoots disturbing leaf recognition. Manual relocation of shoots to grow toward the central region of the part and leaf separation with blue clips was required.

Toward this end, we are in the process of developing a cutting-edge plant phenotyping facility, the “phenome high-throughput investigator (PHI)”, which enables the evaluation of hundreds of traits through non-invasive approaches over time (**Figure 1A**). Our efforts further extend to establishing an operational pipeline for single-leaf-based quantitative phenotypic analyses that allow for the use of this efficient and powerful tool to study leaf senescence and its lifespan. Here, we present our current progress on the establishment of the PHI system and an evaluation of its performance. Moreover, we highlight potential strategies and tactics for phenome-level research toward understanding leaf senescence and lifespan in plants.

## Experimental Scheme: Establishment of a System for Assessment of Physiological Changes in *Arabidopsis* Leaves During Senescence

Leaf senescence is the final stage of the life history of a leaf; thus, all previous experiences prior to the senescence stage can affect senescence and the lifespan process (**Figure 1B-i**). We assessed the morphological and physiological changes occurring during the entire leaf lifespan. In this regard, a quantitative phenotyping system on a single-leaf basis along with age information should be established. Measuring senescence parameters using a mixture of several leaves at a given age of a plant is not a valid analysis of leaf senescence and lifespan because individual leaves of a plant are of different ages (Zentgraf et al., 2004). Leaf developmental events such as senescence can also be modulated by external stresses or exogenous hormones; therefore, kinetic phenotyping analysis in leaves in response to these treatments is an additional valuable approach to dissect responses of leaf senescence (Lim et al., 2007; Schippers et al., 2015).

For the aforementioned purposes, we improved the PHI system to allow the assessment of the imaging-based phenome through single-leaf-based analysis, either in intact plants or detached leaves in 24-well plates (**Figures 1B-ii,iii**). This leaf-based analysis requires a specialized experimental scheme and analytic modules beyond the configuration of a standardized phenotyping system, as detailed below. First, leaf segmentation and tracking in intact plants are necessary for chronological analyses in leaves. Second, a plant mask generated in a RGB image should be transferred and used for analyzing other images (**Figure 1C-i**). This is necessary when the plant signature is indistinguishable from the background soil or pot in a certain image (e.g., fluorescence images in fully senesced leaves). Third, plant trays should be located in the same position at each imaging unit. This could help to segment and track a single leaf of interest from the plants (**Figure 1C-ii**). Lastly, special manipulation is necessary to monitor phenotypes in leaves from the vegetative to senescing stages (**Figure 1C-iii**). Leaves are amenable to mature-stage phenomic analyses; however, the inaccessibility of old leaves covered by new leaves complicates the analyses of chronological events. Thus, leaf separation by placing blue clips on the petiole of the third and fourth leaves at DAE 14 is required for assays of later senescence. In addition, primary or axillary shoots should be directed to grow toward the central region of trays. On the basis of the aforementioned setup, high-throughput phenotypic traits occurring during leaf senescence in *Arabidopsis* would be assessed.

## Proof-Of-Concept: Phenomic Approaches to Evaluate Responses of Senescence in *Arabidopsis* Leaves

Recent advances in non-invasive high-throughput imaging systems have allowed the monitoring of single to hundreds of plant traits to access plant physiological statuses from several thousands of plants in a kinetic manner (Chen et al., 2014; Rahaman et al., 2015; Cabrera-Bosquet et al., 2016). Leaf senescence occurs in an orderly and coordinated manner and involves changes in diverse metabolic processes, including catabolic processes of proteins, lipids, and carbohydrates, along with dismantlement of the photosynthetic apparatus (Lim et al., 2007; Watanabe et al., 2013). Thus, chronological analysis of various biological phenotypic traits is essential for understanding the processes of senescence.

**FIGURE 2:**
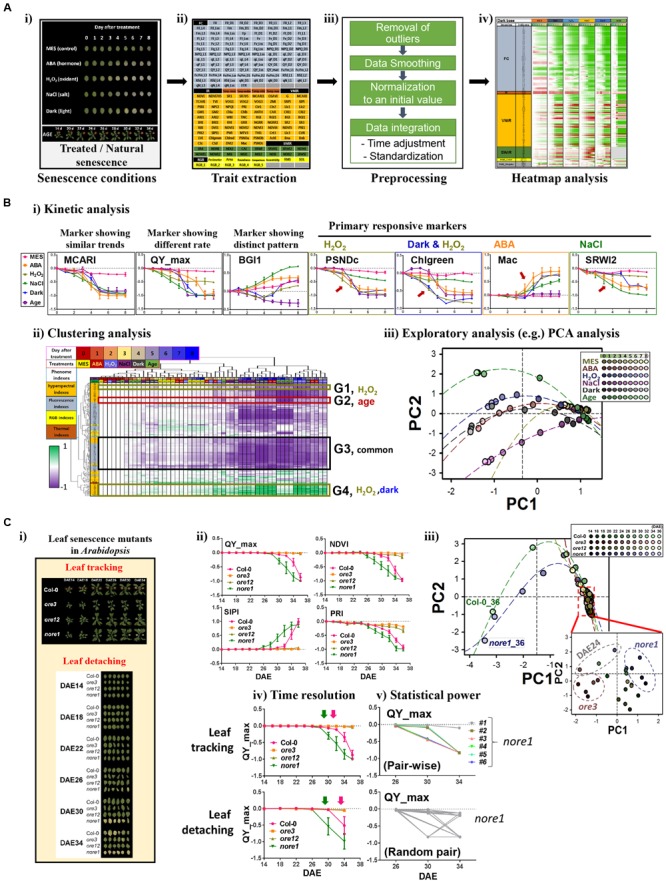
**Phenomic evaluation of leaf senescence responses triggered by various senescence-inducing factors as well as genetic mutations through PHI system. (A,B)** Phenomic evaluation of leaf senescence responses triggered by various senescence-inducing factors and its workflow. The workflow involves trait extraction, preprocessing, heatmap analysis, and data mining. **(A)** Schematic workflow before data mining. **(i)** Picture of representative leaves incubated for indicated days under various senescence conditions such as 50 μM ABA, 15 mM H_2_O_2_, 150 mM NaCl, darkness, or 3 mM MES as a control and the representative plants harboring the third rosette leaf at DAE 14 to 34. **(ii)** Trait extraction, after multimodal imaging, nearly 200 quantitative traits were extracted from thePHI data analyzer. **(iii)** Preprocessing, time-series numeric data were preprocessed by several steps as indicated. **(iv)** Heatmap analysis, temporal profiling of traitsin senescence conditions was visualized using a heatmap for pattern comparison. **(B)** Data mining. Detailed trait and sample analyses were performed by several data mining techniques, including kinetic **(i)**, clustering **(ii)**, and exploratory PCA **(iii)** analyses. In kinetic analyses **(i)**, data represent mean ± SE (*n* = 6). Meanvalue for each genotype or for each age was used for clustering **(ii)** and PCA **(iii)** analysis. MCARI, modified chlorophyll absorption in reflectance index; QY_max, maximum quantum yield of photosystem II; BG1, blue/green index 1; PSNDc, pigment-specific normalized difference; Chlgreen, chlorophyll green index;Mac, Maccioni; SRWI2, simple ratio water index 2. PC, Principal component. The percent variations explained by PC1 and PC2 were 67.2 and 10.1%, respectively. **(C)** PHI system-based phenomic evaluation of senescence responses in well-characterized leaf senescence mutants showing premature or delayed developmental senescence phenotypes. **(i)** Two senescence assays, leaf tracking and leaf detaching, are compared. Shown are the pictures representative of the third leaves ofCol-0, *ore3, ore12*, and *nore1* with different ages from DAE 14 to 34. **(ii)** Kinetic analysis of time-series data of QY_max, NDVI, SIPI, and PRI in wild-type and senescence mutant leaves. NDVI, normalized difference vegetation index; SIPI, structure intensive pigment index; PRI, photochemical reflectance index. Data represent mean ± SE (*n* = 6). **(iii)** PCA-based analysis of senescence progression in wild-type and senescence mutant leaves with different ages. Mean value for each genotype or for each age was used for PCA analysis. The percent variations explained by PC1 and PC2 were 80.1 and 5.7%, respectively. **(iv,v)** Comparison ofsenescence assays in terms of time resolution **(iv)** and statistical power **(v)**. Data represent mean ± SE (*n* = 6).

Here, we explored the limited-scale feasibility of the PHI system for dissecting phenotypic responses during leaf senescence in *Arabidopsis*. System performance using PHI was first evaluated by monitoring the dynamics of phenotypic traits in *Arabidopsis* leaves treated with various senescence-inducing factors such as age, darkness, ABA, one of the stress-related phytohormones, as well as external stresses, including salinity (NaCl) and oxidative stress (H_2_O_2_; **Figure 2A-i**). These phenotypic traits (Supplementary Table S1 and **Figure 2A-ii**) include 208 indices that reflect multiple physiological statuses such as color and growth (12 indices; RGB), metabolic content, vital and vegetative status (77 indices; VNIR), water level or cellular components (16 indices; SWIR), chlorophyll-related photosynthetic performance (99 indices; fluorescence), and water evaporation-based guard cell activity (four indices; infrared). To analyze responses of senescence triggered by various senescence-inducing factors in a comprehensive manner, raw numeric trait datasets should be organized through a preprocessing pipeline that involves (1) removal of outliers, (2) smoothing of time-series data, (3) normalization to the initial value, and (4) data integration throughout time adjustment and data standardization (**Figure 2A-iii**, detailed in Supplementary Information). Such a data integration is necessary for comparative analysis of time-series data with different degrees of effectiveness. An organized and tabled dataset can be displayed in a heatmap for visual summarization and intuitive comparison among different senescence conditions (**Figure 2A-iv**).

Using these datasets, further data mining, including kinetic, clustering, and exploratory analyses, was performed (**Figure 2B**). Kinetic analysis with individual phenotypic traits revealed informative traits for primary or acute responsiveness to each senescence-triggering factor (**Figure 2B-i**). Leaves at different senescing conditions show phenotypic similarity in most of the traits, as represented by a MCARI marker (detailed information of markers in Supplementary Table S1). In contrast, rapid changes of QY_max, a conventional marker reflecting the photochemical quantum efficiency of photosystem II, were observed when treated with H_2_O_2_ and NaCl, implying that QY_max is the effective signature for monitoring responses of leaf senescence to these treatments. This finding also suggests that photosynthetic activity in chloroplasts might be affected as the primary target during senescence, which is consistent with the results of previous transcriptome and metabolome studies (Breeze et al., 2011; Watanabe et al., 2013; Woo et al., 2016). In addition, a distinct temporal pattern in different senescence conditions was observed in some markers such as BGI1. Other markers such as PSNDc, Chlgreen, Mac, and SRWI2 possess a feature of primary responsiveness for H_2_O_2_, both of darkness and H_2_O_2_, ABA, and NaCl, respectively. These traits can further assist in dissecting the temporal progression or coordination of the biological processes related to each condition. More comprehensive relationships among traits and samples can be dissected with further detailed analysis using clustering analysis of phenome-wide data (**Figure 2B-ii**). Although many traits (e.g., belonging to G3) exhibited temporal changes by more than three factors, some groups of traits were associated with specific senescence-inducing factors, including H_2_O_2_ (G1), age (G2), or both dark and H_2_O_2_ (G4). Further detailed and comprehensive sample analysis to dissect their phenotypic relationship requires more sophisticated exploratory statistical techniques such as PCA (**Figure 2B-iii**). PCA indicated that the initial senescence responses, regardless of treatment, were similar among different senescence processes; however, as leaf senescence proceeded over time, the physiological status of leaf senescence caused by different senescence factors changed quite differentially, especially in the case of age and NaCl. It was also shown that dark-induced senescence appeared to be more similar to that of ABA-induced senescence, although senescence responses induced by H_2_O_2_ and darkness shared common markers in the clustering analysis. However, the possibility that different assay conditions among treatments or age interfere with certain reflected or fluorescent lights on the leaves cannot be excluded. Despite some limitations to this study, these results suggest that phenome-wide analyses using a couple of hundreds traits enable us to dissect senescence responses triggered by various senescence inducers.

Next, we further validated the feasibility of this approach by interrogating phenome-based senescence responses in the well-characterized leaf senescence mutants, *oresara 3* (*ore3*), *ore12*, and *not oresara 1* (*nore1*; **Figure 2C-i**). *ore3*, which is allelic to *ethylene insensitive 2*, is insensitive to ethylene signaling, whereas *ore12*, a dominant negative mutant of *ARABIDOPSIS HISTIDINE KINASE 3*, exhibits constitutive cytokinin responses, where both mutations delay leaf senescence (Oh et al., 1997; Kim et al., 2006). In contrast, *nore1* accelerates leaf senescence with enhanced defense response (Lee et al., 2016). The chronological phenomic analyses using a leaf tracking approach were performed at the third and fourth leaves of the wild type (Col) and of these mutant plants from the maturation to senescence stages (from DAE 14 to 36 at 2 days intervals; **Figure 2C-ii**). As previously reported, *ore3* and *ore12* leaves showed delayed senescence phenotypes, whereas *nore1* showed early senescence phenotypes, based on the QY_max value. Although QY_max is widely used as a typical marker of senescence progression, it was found to be less sensitive than other vegetation indices such as NDVI, SIPI, or PRI in VNIR imaging. This finding indicates that reflectance changes due to loss of pigments occur earlier than loss of QY_max during developmental senescence, and these appear to be more useful markers to detect early symptoms of developmental senescence. These kinetic analyses with a few valuable traits may also be evaluated for the progression or rate of senescence responses; *ore3* exhibited a slightly slower change in senescence progression relative to *ore12*. To further explore global changes of phenotypic responses in leaves of these mutants during senescence, PCA analysis was performed for all samples examined with all phenotypic traits (**Figure 2C-iii**). Plotting the individual samples against PC1 and PC2, which collectively explained 86.6% of the variation in samples, afforded a clear separation of Col, early, and delayed senescence mutants at a late senescence stage. Since a slight difference among samples could be masked due to drastic changes in old leaf samples, we further performed PCA analysis to investigate some differences among samples in the maturation to early senescence stages (DAE 14 to 24; **Figure 2C-iii**, embedded graph). From this test, we observed that *nore1* and *ore3* could be distinguished from Col and *ore12*, although visible differences between them were not detected. Interestingly, leaves of Col, *nore1*, and *ore3* from DAE 24 were also resolved from other young leaves, implying that physiological diversity might be explained by the interaction of genetic and developmental factors. Considered together, we conclude that quantitative measurement of phenotypic traits from leaves appears to be important for dissecting leaf senescence, and provides valuable information for phenotypic regulation by senescence-inducing factors or genetic components during senescence.

As the PHI system supports sequential leaf-based analysis from intact plants using leaf tracking, we further addressed advantages of the leaf tracking system by comparison with a conventional leaf detaching assay (**Figure 2C-i**). Practically, the non-invasive phenotyping system requires a much smaller number of plants. In addition, subtle differences among samples could be discerned; temporal analysis could be performed at a higher resolution using leaf tracking (**Figure 2C-iv**), and statistical powers could be increased with a larger number of samples and pair-wise analysis (**Figure 2C-v**). In addition, the performance of the association analysis between traits can be increased, based on the possibility of their one-to-one matching within one sample. Thus, not only is a PHI-based high-throughput system beneficial for performance but it also improves analytical capabilities.

## Conclusion

Here, we developed a specialized high-throughput phenotyping platform for analyzing senescence traits at a single-leaf basis, which will facilitate an alleviation of the phenotyping bottleneck in leaf senescence. As a proof of the concept, we dissected features of various senescence responses through kinetic and PCA analyses utilizing highly resolved and quantitative phenotyping data. In addition, we evaluated advantages of the leaf tracking system in a PHI high-throughput phenotyping system in terms of performance and analytic capabilities. Considered together, we demonstrated the pipeline of phenomics that allows the dissection of a system as complex as leaf lifespan and senescence.

## Perspectives

By virtue of great advances in omics technologies, big data generation has resulted in a major paradigm shift toward data-driven research in plant biology. Along with an increasing feasibility of molecule-based omics, the implementation of automated, high-throughput phenotyping at a similar level will offer new opportunities to understand the complex biological processes occurring in plants (Chen et al., 2014; Granier and Vile, 2014). Our establishment, including the experimental setup and phenotyping data analyses, will open up great opportunities to address concepts and premises that are critical to enhance our fundamental understanding of the as-yet incompletely understood complex process of leaf senescence.

First, our PHI system would allow dynamic, longitudinal, and multi-dimensional analyses that characterize the physiological and regulatory changes along the entire leaf lifespan at a system level. By taking advantage of the PHI system, systematic quantification analyses of all possible traits during the entire leaf development from a large population of plants, including many genetic resources, can be performed. This should result in more detailed insights into mechanisms governing developmental transitions during leaf life history, thereby elucidating important biological principles on how previous developmental programs contribute to the senescence process on a genetic basis. This would also contribute to infer the causal relationship between phenotypic traits at an earlier stage and responses of senescence, which might be valuable for screening during breeding programs. Our pipeline can be extended to the meta-analysis of multiplexed phenotyping data with large-scale quantitative phenotype collections, thereby allowing the depiction of the network relationship from gene- to senescence-related phenotypic traits along the leaf lifespan.

Second, leaf senescence was long believed to be an evolutionarily acquired beneficial process to maximize the fitness of plants. However, no clear evidence has yet emerged linking leaf senescence and fitness. A non-destructive senescence assay and its following fitness measurement such as seed yield will allow the elucidation of their relationship. Furthermore, high-throughput phenotypic analysis of various physiological and developmental traits from the large collection of genetic resources will allow the evaluation of the contribution of each trait to fitness factors, which may thereby elucidate the importance of senescence for fitness, relative to other traits.

Third, a PHI-based high-throughput system supports controlled and precise environmental conditions. This facilitates the investigation of the direct relationship between environmental condition and senescence along with seed yield. It further infers how senescence may contribute to fitness under certain environmental conditions. In addition, high-throughput phenotyping with a large collection of natural accessions under different local simulated climates consisting of photoperiod, light spectrum, temperature, and relative humidity allow the identification of the relationship among senescence, environments, and adaptation to local environmental conditions (Li et al., 2010; Xu, 2016). Combined with genome-wide association analysis, these endeavors will eventually elucidate the mechanisms governing phenotypic plasticity and adaptive mechanisms (Todesco et al., 2010; Brachi et al., 2013; Yang et al., 2014).

Fourth, the main purpose of leaf senescence is the redistribution of nutrients from one part of the plant to another. Thus, senescence can be affected by the removal of sink or neighboring organs, which indicates the existence of inter-organ level coordination (Sekhon et al., 2012). Leaf-based analysis in a PHI system provides favorable tools to dissect inter-organ communication between individual leaves and leaves and other organs such as shoot or root.

Fifth, senescence is regarded as a typical irreversible phenomenon. However, depending on the leaf age and degree of treatments that induce leaf senescence, the primary response of senescence can be recovered. It is feasible to trace back phenotypic changes from leaves with different fates, which might provide some phenotypic clues on how the irreversible onset of senescence is determined.

Phenomic studies can contribute to validate their findings based on transcriptomic, genomic, proteomic, and metabolomic data to senescence processes by providing the outer analytic layer to illustrate collective outputs of dynamic molecular changes such as genes, transcripts, proteins, and metabolites. Thus, combined with these multi-omics data, our phenotyping system is a very promising and valuable tool that allows the investigation of changes to morphology, physiology, and molecular behaviors in a comprehensive manner over leaf lifespan and senescence. This will facilitate an understanding of the mechanisms of life history and senescence over spatial and temporal scales.

## Author Contributions

JL, JK, and PL conceived and designed the experiments. JL, SB, and HC performed the experiments. JL, SB, SJ, and JK analyzed the data. HN provided analysis tools. JK and PL wrote the paper. All authors carefully checked and approved this version of the manuscript.

## Conflict of Interest Statement

The authors declare that the research was conducted in the absence of any commercial or financial relationships that could be construed as a potential conflict of interest.

## Abbreviations

ABA: abscisic acid
BG1: blue/green index 1
Chlgreen: chlorophyll green index
DAE: days after emergence
Mac: Maccioni
MCARI: modified chlorophyll absorption in reflectance index
MES: methyl ester sulfonate
NDVI: normalized difference vegetation index
NORE: not oresara
ORE: oresara
PC: principal component
PCA: principal component analysis
PHI: phenome high-throughput investigator
PRI: photochemical reflectance index
PSNDc: pigment-specific normalized difference
QY_max: maximum quantum yield of photosystem II
RGB: red-green-blue
SE: standard error
SIPI: structure intensive pigment index
SRWI2: simple ratio water index 2
SWIR: shortwave infrared
VNIR: visible and near-infrared

## References

[B1] Brachi B. Villoutreix R. Faure N. Hautekeete N. Piquot Y. Pauwels M. (2013). Investigation of the geographical scale of adaptive phenological variation and its underlying genetics in *Arabidopsis thaliana*. *Mol. Ecol.* 22 4222–4240. 10.1111/mec.12396 23875782

[B2] Breeze E. Harrison E. McHattie S. Hughes L. Hickman R. Hill C. (2011). High-resolution temporal profiling of transcripts during *Arabidopsis* leaf senescence reveals a distinct chronology of processes and regulation. *Plant Cell* 23 873–894. 10.1105/tpc.111.083345 21447789 PMC3082270

[B3] Buchanan-Wollaston V. Page T. Harrison E. Breeze E. Lim P. O. Nam H. G. (2005). Comparative transcriptome analysis reveals significant differences in gene expression and signalling pathways between developmental and dark/starvation-induced senescence in *Arabidopsis*. *Plant J.* 42 567–585. 10.1111/j.1365-313X.2005.02399.x 15860015

[B4] Cabrera-Bosquet L. Fournier C. Brichet N. Welcker C. Suard B. Tardieu F. (2016). High-throughput estimation of incident light, light interception and radiation-use efficiency of thousands of plants in a phenotyping platform. *New Phytol.* 212 269–281. 10.1111/nph.14027 27258481

[B5] Chen D. Neumann K. Friedel S. Kilian B. Chen M. Altmann T. (2014). Dissecting the phenotypic components of crop plant growth and drought responses based on high-throughput image analysis. *Plant Cell* 26 4636–4655. 10.1105/tpc.114.129601 25501589 PMC4311194

[B6] Crowell S. Falcao A. X. Shah A. Wilson Z. Greenberg A. J. McCouch S. R. (2014). High-resolution inflorescence phenotyping using a novel image-analysis pipeline, PANorama. *Plant Physiol.* 165 479–495. 10.1104/pp.114.238626 24696519 PMC4044845

[B7] Furbank R. T. Tester M. (2011). Phenomics-technologies to relieve the phenotyping bottleneck. *Trends Plant Sci.* 16 635–644. 10.1016/j.tplants.2011.09.005 22074787

[B8] Granier C. Vile D. (2014). Phenotyping and beyond: modelling the relationships between traits. *Curr. Opin. Plant Biol.* 18 96–102. 10.1016/j.pbi.2014.02.009 24637194

[B9] Guo Y. (2013). Towards systems biological understanding of leaf senescence. *Plant Mol. Biol.* 82 519–528. 10.1007/s11103-012-9974-2 23065109

[B10] Humplík J. F. Lazár D. Husičková A. Spíchal L. (2015). Automated phenotyping of plant shoots using imaging methods for analysis of plant stress responses – a review. *Plant Methods* 11 1–10. 10.1186/s13007-015-0072-8 25904970 PMC4406171

[B11] Kim H. J. Ryu H. Hong S. H. Woo H. R. Lim P. O. Lee I. C. (2006). Cytokinin-mediated control of leaf longevity by AHK3 through phosphorylation of ARR2 in *Arabidopsis*. *Proc. Natl. Acad. Sci. U.S.A.* 103 814–819. 10.1073/pnas.0505150103 16407152 PMC1334631

[B12] Kim J. Woo H. R. Nam H. G. (2016). Toward systems understanding of leaf senescence: an integrated multi-omics perspective on leaf senescence research. *Mol. Plant* 9 813–825. 10.1016/j.molp.2016.04.017 27174403

[B13] Lee I. H. Lee I. C. Kim J. Kim J. H. Chung E. H. Kim H. J. (2016). NORE1/SAUL1 integrates temperature dependent defense programs involving SGT1b and PAD4 pathways and leaf senescence in *Arabidopsis*. *Physiol. Plant.* 158 180–199. 10.1111/ppl.12434 26910207

[B14] Li L. H. Xing Y. F. Chang D. Fang S. S. Cui B. Y. Li Q. (2016). CaM/BAG5/Hsc70 signaling complex dynamically regulates leaf senescence. *Sci. Rep.* 6:31889 10.1038/Srep31889 PMC4990970 27539741

[B15] Li Y. Huang Y. Bergelson J. Nordborg M. Borevitz J. O. (2010). Association mapping of local climate-sensitive quantitative trait loci in *Arabidopsis thaliana*. *Proc. Natl. Acad. Sci. U.S.A.* 107 21199–21204. 10.1073/pnas.1007431107 21078970 PMC3000268

[B16] Liebsch D. Keech O. (2016). Dark-induced leaf senescence: new insights into a complex light-dependent regulatory pathway. *New Phytol.* 212 563–570. 10.1111/nph.14217 27716940

[B17] Lim P. O. Kim H. J. Nam H. G. (2007). Leaf senescence. *Annu. Rev. Plant Biol.* 58 115–136. 10.1146/annurev.arplant.57.032905.105316 17177638

[B18] Mochida K. Shinozaki K. (2011). Advances in omics and bioinformatics tools for systems analyses of plant functions. *Plant Cell Physiol.* 52 2017–2038. 10.1093/pcp/pcr153 22156726 PMC3233218

[B19] Oh S. A. Park J. H. Lee G. I. Paek K. H. Park S. K. Nam H. G. (1997). Identification of three genetic loci controlling leaf senescence in *Arabidopsis thaliana*. *Plant J.* 12 527–535. 9351240 10.1046/j.1365-313x.1997.00527.x

[B20] Rahaman M. M. Chen D. Gillani Z. Klukas C. Chen M. (2015). Advanced phenotyping and phenotype data analysis for the study of plant growth and development. *Front. Plant Sci.* 6:619 10.3389/fpls.2015.00619 PMC4530591 26322060

[B21] Rajasundaram D. Selbig J. (2016). More effort - more results: recent advances in integrative ’omics’ data analysis. *Curr. Opin. Plant Biol.* 30 57–61. 10.1016/j.pbi.2015.12.010 26890084

[B22] Schippers J. H. (2015). Transcriptional networks in leaf senescence. *Curr. Opin. Plant Biol.* 27 77–83. 10.1016/j.pbi.2015.06.018 26190740

[B23] Schippers J. H. Schmidt R. Wagstaff C. Jing H. C. (2015). Living to die and dying to live: the survival strategy behind leaf senescence. *Plant Physiol.* 169 914–930. 10.1104/pp.15.00498 26276844 PMC4587445

[B24] Sekhon R. S. Childs K. L. Santoro N. Foster C. E. Buell C. R. de Leon N. (2012). Transcriptional and metabolic analysis of senescence induced by preventing pollination in maize. *Plant Physiol.* 159 1730–1744. 10.1104/pp.112.199224 22732243 PMC3425209

[B25] Slovak R. Göschl C. Su X. Shimotani K. Shiina T. Busch W. (2014). A scalable open-source pipeline for large-scale root phenotyping of *Arabidopsis*. *Plant Cell* 26 2390–2403. 10.1105/tpc.114.124032 24920330 PMC4114940

[B26] Thomas H. (2013). Senescence, ageing and death of the whole plant. *New Phytol.* 197 696–711. 10.1111/nph.12047 23176101

[B27] Todesco M. Balasubramanian S. Hu T. T. Traw M. B. Horton M. Epple P. (2010). Natural allelic variation underlying a major fitness trade-off in *Arabidopsis thaliana*. *Nature* 465 632–636. 10.1038/nature09083 20520716 PMC3055268

[B28] Topp C. N. Iyer-Pascuzzi A. S. Anderson J. T. Lee C. R. Zurek P. R. Symonova O. (2013). 3D phenotyping and quantitative trait locus mapping identify core regions of the rice genome controlling root architecture. *Proc. Natl. Acad. Sci. U.S.A.* 110 E1695–E1704. 10.1073/pnas.1304354110 23580618 PMC3645568

[B29] Watanabe M. Balazadeh S. Tohge T. Erban A. Giavalisco P. Kopka J. (2013). Comprehensive dissection of spatiotemporal metabolic shifts in primary, secondary, and lipid metabolism during developmental senescence in *arabidopsis*. *Plant Physiol.* 162 1290–1310. 10.1104/pp.113.217380 23696093 PMC3707545

[B30] Woo H. R. Koo H. J. Kim J. Jeong H. Yang J. O. Lee I. H. (2016). Programming of plant leaf senescence with temporal and inter-organellar coordination of transcriptome in *Arabidopsis*. *Plant Physiol.* 171 452–467. 10.1104/pp.15.01929 26966169 PMC4854694

[B31] Xu Y. (2016). Envirotyping for deciphering environmental impacts on crop plants. *Theor. Appl. Genet.* 129 653–673. 10.1007/s00122-016-2691-5 26932121 PMC4799247

[B32] Yang W. Duan L. Chen G. Xiong L. Liu Q. (2013). Plant phenomics and high-throughput phenotyping: accelerating rice functional genomics using multidisciplinary technologies. *Curr. Opin. Plant Biol.* 16 180–187. 10.1016/j.pbi.2013.03.005 23578473

[B33] Yang W. Guo Z. Huang C. Duan L. Chen G. Jiang N. (2014). Combining high-throughput phenotyping and genome-wide association studies to reveal natural genetic variation in rice. *Nat. Commun.* 5:5087 10.1038/ncomms6087 PMC4214417 25295980

[B34] Zentgraf U. Jobst J. Kolb D. Rentsch D. (2004). Senescence-related gene expression profiles of rosette leaves of *Arabidopsis thaliana*: leaf age versus plant age. *Plant Biol.* 6 178–183. 10.1055/s-2004-815735 15045669

