# Supplementary material for: High-Throughput and Computational Study of Leaf Senescence through a Phenomic Approach
Source: Front Plant Sci. 2017 Feb 23;8:250. doi: 10.3389/fpls.2017.00250 (PMC5322180; doi:10.3389/fpls.2017.00250)
Supplement: Supplementary file 2 [file Supplementary_Information.docx]

Supplementary Information

**High-throughput and computational study of leaf senescence through a phenomic approach**

Jae Il Lyu^1#^, Seung Hee Baek^2#^, Sukjoon Jung^2^, Hyosub Chu^1^, Hong Gil Nam^1,2^, Jeongsik Kim^1!^, and Pyung Ok Lim^2!^

# These authors contributed equally to this work.

! Co-corresponding authors.

Email: yorus@postech.ac.kr (J. Kim) and polim@dgist.ac.kr (P.O. Lim)

**This file includes:**

Supplementary materials and methods

Supplementary reference

**Other supplementary information for this manuscript includes the following:**

Supplementary Table S1 – The additional table is in the form of excel file.

SUPPLEMENTARY MATERIALS AND METHODS

**Plant cultivations and senescence assays**

*Arabidopsis* seeds were sown in pots (60 mm × 60 mm × 90 mm, T68S, Garden City Plastics) filled with steam-sterilized soil mixture (a 1:1:1 mix of vermiculite, perlite, and peat moss) after seed imbibition (4 °C for 3 days). A non-woven cover was used as a soil cover. Seedlings (n = 40) were grown in environmentally controlled growth rooms (a part of the phenome high-throughput investigator [PHI] system) with a 16 h light/8 h dark cycle at 23 °C, relative humidity of 60%, and light-emitting diode (LED) white light of 90–100 µmol m^-2^ s^-1^ supplemented with red light of 4 µmol m^-2^ s^-1^ and far-red light of 0.6 µmol m^-2^ s^-1^. Pots (n = 12) with seedlings of similar size were placed along the outer region of a tray (5 × 4 pots per tray). Plants were watered twice a week with 10 mL of tap water using an automatic and programmed supply system. To access phenotypic traits on the leaves using leaf tracking during developmental senescence, plastic clips as a leaf separation tool were placed above the petiole parts of third and fourth leaves (14 days after leaf emergence; DAE) from 23-day-old plants (Figure 1C-iii). The main and axillary shoots of plants were placed toward the central empty region of trays using curved wires. Images were taken in two-day intervals until the 3^rd^ and 4^th^ leaves were completely yellow. In comparison with the leaf tracking approaches, developmental senescence responses were also monitored using detached leaves harvested at indicated ages from plants that were grown in an environmentally controlled room with similar conditions, but using fluorescent light of 150 µmol m^-2^ s^-1^. For stress- or hormone-induced senescence assays, leaves were detached from 23-day-old-plants and transferred into 24-well plates containing 3 mM MES with mock, stress-inducing chemicals (15 mM H_2_O_2_ and 150 mM NaCl) or hormones (50 µM abscisic acid [ABA]). Plates were kept in the growth rooms of the PHI system (16 h light/ 8 h dark cycle at 23 °C) and their imaging was performed daily. For dark-induced senescence, plates were stored as with other assays; however, they were wrapped with aluminum foil and uncovered during their imaging.

**Phenome high-throughput investigator (PHI)**

PHI is an automated high-throughput imaging system coupled with a precise cultivation system (Plantscreen^TM^, Photons Systems Instruments; Figure 1A, i and ii). Two plant growth rooms support cultivation of different types of plants or the same type of plants under different environmental conditions that are tightly controlled, such as light intensity (up to ~1,000 µmol photons m^-2^ s^-1^), CO_2_ concentration (300 ppm to 3,000 ppm), temperature (4 °C–40 °C), and relative humidity (30%–99%). The PHI supports analysis for two different types of trays: small (20 pots per one tray) and big pots (two pots per one tray). Up to 21 trays can be transferred from the growth room to the acclimation tunnel for dark/light adaptation, and then moved to imaging units through a conveyor system following a predefined schedule and protocols or by a call by the user to the operating software. The fertilizer and/or chemicals can be supplied by the automatic and programmed supply system. The image station is equipped with five non-invasive camera-based cabinet imaging units: fluorescence, RGB (top and side view [line scanner]), infrared, hyperspectral imaging (visible and near-infrared [VNIR]; 400 nm to 1,000 nm, shortwave infrared [SWIR]; 1,000 nm to 2,500 nm) and three-dimensional (3D) imaging units. Fluorescence, infrared, and hyperspectral images were captured from the top view only. All of the images were processed on the shoot of a plant. A total time of approximately 2 h was required in order to measure 12 trays (240 plants for small pots or 24 plants for large pots) under a standard imaging protocol using all of the imaging units. The error rate of the instruments during the data acquisition was less than 0.5 %. The raw images and processed numeric data are stored in a database on site and can be displayed graphically or in a tabular format when requested in the plantscreen^TM^ data analyzer (Ver. 3.1.0.9, PSI; Figure 1A-iii). Those data can be exported as a TAR file for the fluorescence image, a PNG file for the RGB image, a RAW file for the thermal image, a BIL file for the hyperspectral image, and a PCD file for the 3D image. No significant negative side effects were observed in terms of plant growth when data with and without fluorescence, thermal, and hyperspectral imaging were compared.

**Image acquisition and time series data analysis**

Image acquisition was performed using five cameras for fluorescence, top-view red-blue-green (RGB), thermal, and hyperspectral (VNIR and SWIR) imaging, according to manufacturer’s instructions or Awlia et al. (2016) at 2 h and 6 h after lights on for *in planta* leaf tracking and for the detached leaf assay, respectively. Chlorophyll fluorescence imaging was performed using the quenching kinetics protocol after 15 min dark adaptation. All raw image data and quantitative trait data were collected during imaging acquisition, but the data were re-analyzed by manual curation when the Plantscreen data analyzer was unable to perform plant recognition to a satisfactory level. The collected time series data were preprocessed for proper analysis, which includes 1) removal or recalculation of outliers, 2) smoothing of time-series data for missing data or outliers, 3) normalization to the initial value, and 4) data integration. For data integration, temporal data were normalized by dividing the difference between the maximum and minimum values of the time-series data for each trait. Outliers were defined as values smaller or larger than three standard deviations from the median of the time-series data or biological samples at each time for each parameter set. In addition, owing to the high intensity of the background signals, any negative values were also considered as outliers. Missing data were imputed using the mean of temporally adjacent observed values.

To compare kinetic patterns of trait values among different senescence-inducing factors, the time scale of time-series data was adjusted as detailed below. Based on the total phenome profiles during senescence, the time to reach 50% of the difference between the maximum and minimum values of the temporal data was calculated for individual traits, and their median time values (MTVs) were used to predict the effectiveness of the treatment. The original time-series data were fitted to Sigmoid curves:

$f(x)=a\frac{1}{1+e^{-k\left( x-x_{o} \right)}}+b$ (Equation 1)

and their timescale was adjusted by the ratio of the MTVs of each treatment to the MTV of the control (*i.e.*, the dark treatment). Values at the indicated time were estimated using the created sigmoid function. Inter-day or inter-assay comparability was ensured by adjustment of time-series data by considering MTV values as described above. The data variability of the groups and inter-individual differences were shown as the mean values and error bars (SE), respectively. All data mining was performed using SPSS statistics 20. A hierarchical clustering analysis was performed using the Euclidean distance as the similarity measure and complete linkage for the agglomerative method. The PCA was performed using the Pearson’s correlation matrix.

SUPPLEMENTARY REFERENCE

Awlia, M., Nigro, A., Fajkus, J., Schmoeckel, S.M., Negrao, S., Santelia, D., et al. (2016). High-throughput non-destructive phenotyping of traits that contribute to salinity tolerance in *Arabidopsis thaliana*. *Front. Plant Sci.* 7**,** 1414. doi: 10.3389/fpls.2016.01414
